# Direct Generation of Immortalized Erythroid Progenitor Cell Lines from Peripheral Blood Mononuclear Cells

**DOI:** 10.3390/cells10030523

**Published:** 2021-03-01

**Authors:** Abhirup Bagchi, Aneesha Nath, Vasanth Thamodaran, Smitha Ijee, Dhavapriya Palani, Vignesh Rajendiran, Vigneshwaran Venkatesan, Phaneendra Datari, Aswin Anand Pai, Nancy Beryl Janet, Poonkuzhali Balasubramanian, Yukio Nakamura, Alok Srivastava, Kumarasamypet Murugesan Mohankumar, Saravanabhavan Thangavel, Shaji R. Velayudhan

**Affiliations:** 1 Center for Stem Cell Research (A Unit of InStem, Bengaluru, India), Christian Medical College, Vellore 632002, Tamil Nadu, India; abhirup@cmcvellore.ac.in (A.B.); aneesha.micro@gmail.com (A.N.); vasanthbiot@gmail.com (V.T.); smitha.ijee@cmcvellore.ac.in (S.I.); grlab@cmcvellore.ac.in (D.P.); vignesh.r@cmcvellore.ac.in (V.R.); vigneshwaran.v@cmcvellore.ac.in (V.V.); aloks@cmcvellore.ac.in (A.S.); mohankumarkm@cmcvellore.ac.in (K.M.M.); sthangavel@cmcvellore.ac.in (S.T.); 2 Manipal Academy of Higher Education, Manipal 576104, Karnataka, India; 3 Department of Hematology, Christian Medical College, Vellore 632002, Tamil Nadu, India; drphaneendradatari@outlook.com (P.D.); aswin.anand@cmcvellore.ac.in (A.A.P.); nancyarthur@cmcvellore.ac.in (N.B.J.); bpoonkuzhali@cmcvellore.ac.in (P.B.); 4 Cell Engineering Division, RIKEN BioResource Research Center, Ibaraki 3050074, Japan; yukionak@brc.riken.jp

**Keywords:** erythroid, differentiation, immortalization, peripheral blood, HPV E6/E7

## Abstract

Reliable human erythroid progenitor cell (EPC) lines that can differentiate to the later stages of erythropoiesis are important cellular models for studying molecular mechanisms of human erythropoiesis in normal and pathological conditions. Two immortalized erythroid progenitor cells (iEPCs), HUDEP-2 and BEL-A, generated from CD34^+^ hematopoietic progenitors by the doxycycline (dox) inducible expression of human papillomavirus E6 and E7 (HEE) genes, are currently being used extensively to study transcriptional regulation of human erythropoiesis and identify novel therapeutic targets for red cell diseases. However, the generation of iEPCs from patients with red cell diseases is challenging as obtaining a sufficient number of CD34^+^ cells require bone marrow aspiration or their mobilization to peripheral blood using drugs. This study established a protocol for culturing early-stage EPCs from peripheral blood (PB) and their immortalization by expressing HEE genes. We generated two iEPCs, PBiEPC-1 and PBiEPC-2, from the peripheral blood mononuclear cells (PBMNCs) of two healthy donors. These cell lines showed stable doubling times with the properties of erythroid progenitors. PBiEPC-1 showed robust terminal differentiation with high enucleation efficiency, and it could be successfully gene manipulated by gene knockdown and knockout strategies with high efficiencies without affecting its differentiation. This protocol is suitable for generating a bank of iEPCs from patients with rare red cell genetic disorders for studying disease mechanisms and drug discovery.

## 1. Introduction

Human erythropoiesis is a multistep process that involves successive differentiation of hematopoietic stem cells (HSCs) into erythroid progenitors/precursors, reticulocytes, and red blood cells (RBCs). Several genetic diseases affect the differentiation, proliferation, and normal physiological functions of erythroid cells. Sickle cell disease (SCD) and β-thalassemia, two common genetic diseases, affect red cell development and hemoglobinization [1]. Diamond Blackfan anemia (DBA) and congenital dyserythropoietic anemia (CDA) affect normal erythropoiesis due to deficiencies in important molecular processes in erythroid cells [2,3]. For understanding the molecular basis of normal versus pathological erythropoiesis, erythroid cells generated by in vitro erythropoiesis by differentiating primary CD34^+^ hematopoietic stem and progenitor cells (HSPCs) are commonly being used [4,5,6,7,8,9]. The main challenge in this approach is the requirement of multiple donations of HSPCs from a single individual to generate adequate numbers of erythroid cells for the downstream experiments. For many diseases, obtaining HSPCs by their mobilization using drugs is impossible due to the pathology of the diseases. Although induced pluripotent stem cells (iPSCs) generated from patients with erythroid diseases serve as a stable source of erythroid cells [10,11], this strategy is tedious and expensive. Furthermore, erythroid cells generated from iPSCs exhibit characteristics of primitive erythrocytes [12,13] with inefficient terminal maturation [14,15,16] and expression of embryonic and fetal stage proteins [13,14,15,16,17]. Generation of mouse models for human diseases is time-consuming and expensive, and some human red cell diseases are not accurately mimicked in the mouse models [18].

Recent studies have shown that immortalized erythroid progenitor cells (iEPCs) could be generated by the lentiviral expression of HPV16 E6/E7 (HEE) genes in the erythroid progenitor cells (EPCs) differentiated from CD34^+^ HSPCs [19,20]. iEPCs provide an unlimited supply of erythroid cells, and they enable in vitro manipulation of molecular targets to study mechanisms of erythropoiesis [21,22,23,24]. Two such iEPCs, HUDEP-2 [19] and BEL-A [20], derived from cord blood and adult HSPCs, respectively, are currently available for research applications. HUDEP-2 has been extensively used in basic research to understand the mechanisms of erythropoiesis [25,26,27] and for validating the therapeutical targets for hemoglobinopathies by gene manipulation using RNA interference (RNAi) or gene editing [21,23,26,28,29,30,31]. BEL-A was used as a stable source of reticulocytes to study host-parasite interaction in *Plasmodium falciparum* invasion [32]. This line was also engineered by gene-editing to produce enucleated RBCs with enhanced transfusion compatibility [33]. Recently, an iEPC was generated using a similar approach from the HSPCs of a patient with HbE/β-thalassemia for disease modeling [34].

Peripheral blood (PB) contains a small number of highly proliferative blast forming unit-erythroid (BFU-E) erythroid progenitors [35,36,37], which can be expanded and differentiated in culture [38,39,40,41,42,43]. Generation of iEPCs from the PB-EPCs will be extremely valuable for disease modeling as it will avoid the challenges involved in obtaining CD34^+^ HSPCs from patients. We have successfully generated iEPCs from PB (PBiEPCs), and they have been extensively characterized for their morphology and differentiation and hemoglobinization potentials. One of the cell lines was found to be superior to HUDEP-2 and comparable to BEL-A for its enucleation potential. The PBiEPCs were also found to be highly amenable for gene manipulations by lentiviral RNAi and clustered regularly interspaced short palindromic repeats (CRISPR)/Cas9 mediated gene editing. We also describe the kinetics of the immortalization process and the associated technical challenges that need to be overcome for the successful immortalization of erythroid cells.

## 2. Materials and Methods

### 2.1. Production of Lentiviruses

HEK-293T cells were cultured in Dulbecco’s modified Eagle medium (DMEM) supplemented with 10% fetal bovine serum (FBS), 2 mM l-glutamine, 100 U/mL penicillin, and 100 μg/mL streptomycin (all the reagents were purchased from Thermo Fisher Scientific, Inc., Grand Island, NY, USA). For the preparation of lentiviruses, HEK-293T cells were transfected with TRE-HEE-UbC-hKO1-rtTA (CSIV-TRE-HEE-UbC-KT) encoding HPV16-E6/E7 (HEE) genes (a kind gift from Yukio Nakamura) [19] and lentiviral envelope plasmid pMD2.G (Addgene 12259) and the packaging plasmid psPAX2 (Addgene 12260) (gifts from Didier Trono) using the Trans-IT LT1 transfection reagent (Mirus Bio LLC, Madison, WI, USA), following the manufacturer’s protocols. The viral supernatants were collected at 48, 60, and 72 h, pooled and concentrated 100 times after ultracentrifugation, and aliquoted and frozen at −80 °C.

### 2.2. Generation of iEPCs from Peripheral Blood Mononuclear Cells (PBMNCs)

PBMNCs were isolated from normal healthy donors using Ficoll-Paque (GE Healthcare, Uppsala, Sweden). For the expansion of EPCs, PBMNCs were cultured for 24 h in the erythroid progenitor medium (EPM) containing StemSpan SFEM-II (StemCell Technologies, Vancouver, BC, Canada) supplemented with 3 U/mL erythropoietin (Epo) (Peprotech Inc., Rocky Hill, NJ, USA), 50 ng/mL stem cell factor (SCF) (ImmunoTools GmbH, Friesoythe, Lower Saxony, Germany), 10 ng/mL interleukin-3 (IL-3) (ImmunoTools GmbH), 40 ng/mL insulin growth factor-1 (IGF-1) (ImmunoTools GmbH), 1 μM dexamethasone (Sigma-Aldrich, St. Louis, MO, USA), 2 mM l-glutamine, 100 U/mL penicillin, and 100 μg/mL streptomycin. About two million cells were transduced with TRE-HEE-UbC-hKO1-rtTA lentiviruses by spinfection at 2250 rpm for 1.5 h and were cultured in EPM, with half medium change on every alternative day. From the fourth day, the cells were supplemented with 1 μg/mL doxycycline (dox) (Sigma-Aldrich, St. Louis, MO, USA). Throughout the culture, the cells were maintained at a density of 5–6 × 10^5^ cells/mL at 37 °C, 5% CO_2_ with complete medium change on alternative days.

### 2.3. Generation of iEPCs from CD34^+^ HSPCs

CD34^+^ HSPCs were isolated using a magnetic bead-based positive selection kit (StemCell Technologies, Vancouver, BC, Canada) from the PBMNCs of a hematopoietic stem cell donor. The purified CD34^+^ cells were cultured in HSPC expansion medium composed of StemPro-34 SFM (Thermo Fisher Scientific, Grand Island, NY, USA) containing 100 ng/mL SCF, 100 ng/mL FLT3-Ligand (FLT3-L) (ImmunoTools GmbH), 20 ng/mL IL-6 (ImmunoTools GmbH), 20 ng/mL IL-3, 100 U/mL penicillin, and 100 μg/mL streptomycin. Two days after initiating the culture, the cells were transduced with TRE-HEE-UbC-hKO1-rtTA lentiviruses by spinfection at 2250 rpm for 1.5 h. After four days, the cells were transferred to EPM containing 1 µg/mL of dox. Throughout the culture, the cells were maintained at a density of 5–6 × 10^5^ cells per mL of the medium at 37 °C, 5% CO_2,_ with complete medium change on alternative days.

### 2.4. Differentiation of iEPCs

For induction of the erythroid differentiation of iEPCs, a previously described protocol was used with minor modifications [33]. Briefly, iEPCs cultured in EPM were seeded at a density of 2 × 10^5^ cells/mL in erythroid differentiation medium I (EDM I) for two days. EDM I consisted of Iscove’s Modified Dulbecco’s Medium (IMDM) containing Glutamax (ThermoFisher Scientific, Grand Island, NY, USA), 3% human AB serum (MP Biomedicals, Solon, OH, USA), 2% Fetal Bovine Serum (ThermoFisher Scientific, Grand Island, NY, USA), 200 µg/mL holotransferrin (Sigma-Aldrich, St. Louis, MO, USA), 3 U/mL heparin (Sigma-Aldrich, St. Louis, MO, USA), 10 μg/mL insulin (Sigma-Aldrich, St. Louis, MO, USA), 3 U/mL Epo (Peprotech Inc.), 10 ng/mL SCF (Immunotools GmbH), 1 ng/mL IL-3 (Immunotools GmbH), 1 µg/mL dox, 100 U/mL penicillin, and 100 μg/mL streptomycin. On day 2, the cells were reseeded at a density of 3 × 10^5^ cells/mL in EDM-I and cultured for two days. On day 4, the cells were seeded at a density of 5 × 10^5^ cells/mL in EDM-II (EDM I without dox) and cultured for two days. On day 6, the cells were seeded at a density of 1 × 10^6^ cells/mL in EDM-III (EDM II with 500 µg/mL holotransferrin) for two days, and on day 8, the cells were reseeded at 1 × 10^6^ cells/mL in EDM-IV (EDM III without SCF and IL-3) until the end of differentiation with medium change on every alternative day.

### 2.5. Morphology Analysis and Staining

Approximately 0.5 × 10^5^ EPCs were washed with phosphate-buffered saline (PBS). Cell smears were prepared on glass slides using Cytospin 3 (Thermo Fisher Scientific, Waltham, MA, USA) and were fixed with methanol and then stained with Giemsa stain (Sigma-Aldrich, St. Louis, MO, USA), following the manufacturer’s protocol. Cell morphology was visualized under a light microscope.

### 2.6. Flow Cytometry

A sample of 10^5^ cultured erythroid cells was washed with PBS and suspended in 100 µL of PBS containing anti-CD71-FITC (dilution 1:50) and anti-CD235a PE-Cy7 (dilution 1:50) or anti CD235a Bv421 (dilution 1:50) antibodies (BD Pharmingen, San Jose, CA, USA). The cells were incubated with the antibodies in the dark for 20 min, washed with PBS, and then analyzed on the FACS AriaIII flow cytometer (BD Biosciences, San Jose, CA, USA) or Cytoflex LX Flow Cytometer (Beckmann Coulter, Indianapolis, IN, USA). To analyze F-cells (HbF expressing erythroid cells), the cultured erythroid cells from day 6 in EDM III were harvested, washed with PBS/0.1% BSA, and fixed in PBS/0.1% BSA containing 0.05% glutaraldehyde (MP Biomedicals, Solon, OH, USA). The fixed cells were permeabilized with PBS/0.1% BSA containing 0.01% Triton-X-100 (Sigma Aldrich, St. Louis, MO, USA) and stained with anti-HbF APC antibody (dilution 1:25) (Invitrogen Corporation, Camarillo, CA, USA). The flow cytometry analysis was performed in a BD AriaIII flow cytometer (BD Biosciences, San Jose, CA, USA).

### 2.7. Karyotyping

Karyotyping of the iEPCs was performed using standard protocols. Briefly, 2 × 10^6^ cells were treated with 200 μg/mL colcemid (Life Technologies, Grand Island, NY, USA) for 20 min. The cells were centrifuged and resuspended in a hypotonic solution (0.075 M potassium chloride) for 12 min at 37 °C, and the cells were fixed with Carnoy’s fixative. Chromosome spreads were made and stained with Leishman’s stain (Sigma Aldrich, St. Louis, MO, USA). G-banded metaphases were analyzed using an AxioImager A1 microscope (Carl Zeiss Inc., Thornwood, NY, USA) and Ikaros Software (Metasystems GmbH, Altlußheim, Germany).

### 2.8. High-Performance Liquid Chromatography (HPLC) for Globin Chain Analysis

Globin chain analysis was performed using a previously reported method [44], with minor modifications. Briefly, 3 million differentiated iEPCs were resuspended in 100 µL of distilled water and frozen and thawed thrice in −80 °C. The cell lysate was centrifuged at 14,000× *g* for 10 min at 4 °C, and the supernatant was transferred to vials for injection into the HPLC system. Globin chains were quantified using a Shimadzu UFLC consisting of binary gradient pumps, an autosampler, and a column oven coupled with UV detection (all equipment from Shimadzu, Kyoto, Japan), and the data were analyzed using LC Solutions software (Shimadzu, Kyoto, Japan). Chromatographic separation of the analytes was done using an Aeris Widepore 3.6 lm XB-C18 25 cm, 4.6 mm column behind a Security Guard UHPLC Widepore C18 4.6 mm guard column (Phenomenex, Torrance, CA, USA). The gradient method was used for elution with mobile phases (Solvent A-0.1% trifluoroacetic acid (TFA, Sigma-Aldrich, St. Louis, MO, USA), pH 3.0, and 40% Solvent B-0.1% TFA in acetonitrile (Sigma-Aldrich, St. Louis, MO, USA)) at a flow rate of 1.0 mL/min and column temperature maintained at 70 °C. The total run time was eight minutes. The UV detection was set at 190 nm for globin chain detection.

### 2.9. Knockdown of BCL11A in iEPCs

The MND (myeloproliferative sarcoma virus enhancer, negative control region deleted, dl587rev primer binding site substituted) promoter sequence was PCR amplified from PTRip-MND-GFP (a gift from Francoise Pflumio) and cloned into pZIP-hCMV-ZsGreen-Puro lentiviral vector (Transomics technologies Inc.) using *Cla*I and *Age*I restriction sites to generate pZIP-MND-ZsGreen-Puro. Short hairpin RNAs (shRNAs) against B-cell lymphoma/leukemia 11A (*BCL11A*) gene were obtained from the Sherwood shRNA design algorithm [45]. The shRNA oligo was amplified and cloned into *Hpa*I digested pZIP-MND-ZsGreen-Puro using the NEBuilder Hi-fi DNA assembly cloning kit (NEB Biolabs Inc., Ipswich, MA, USA) to generate the pZIP-MND-ZsGreen-Puro-shBCL11A plasmid. PBiEPC-1, PBiEPC-2, and CD34iEPC were transduced with the lentiviruses in the presence of 8 µg/mL polybrene (Sigma-Aldrich, St. Louis, MO, USA). After five days, the ZsGreen^+^ cells were sorted using a BD FACS Aria III flow sorter (BD Biosciences). *BCL11A* knockdown was analyzed by western blot and qPCR in the undifferentiated PBiEPC-1 cells. Fetal hemoglobin (HbF) and γ-globin levels were measured in the differentiated PBiEPC-1 using flow cytometry and HPLC, respectively.

### 2.10. CRISPR/Cas9 Based Gene Editing of BCL11A Exon 2 and Enhancer Regions

gRNAs targeting *BCL11A* exon 2 and enhancer regions were designed using the CRISPR Design Tool [46] and CHOPCHOP [47]. The most specific and efficient gRNAs (Table S1) were synthesized with modifications to improve RNA stability (Synthego Corp., Redwood City, CA, USA). Ribonucleoprotein (RNP) complexes containing a 2:1 ratio of each gRNA and Cas9 protein (Takara Bio Inc., Kusatsu, Shiga, Japan) were incubated for 10 min at room temperature and used for electroporation. A total of 2 × 10^5^ cells were suspended in 20 µL of electroporation buffer P3 (Lonza, Basel, Switzerland)), and electroporation was performed using Lonza 4D nucleofector (Lonza, Basel, Switzerland) and the program DZ100. After electroporation, the cells were suspended in 100 μL of pre-warmed medium without antibiotics, incubated for 10 min, and then transferred to cell culture plates. After five days, PCRs were carried out to amplify the genomic regions flanking the gRNA binding regions, followed by Sanger DNA sequencing of the amplicons using specific primers (Table S1) and Inference of CRISPR Edits (ICE) analysis [48] to calculate the percentages of the mutations by gene editing.

### 2.11. Quantitative Real-Time PCR Analysis

Total RNA was extracted from iEPCs using RNAiso Plus (Takara Bio Inc., Kusatsu, Shiga, Japan). One μg of total RNA was used for reverse transcription using the Primescript RT reagent kit (Takara Bio Inc., Kusatsu, Shiga, Japan) using the manufacturer’s instructions. Quantitative RT-PCR was set up with SYBR Premix Ex Taq II (Takara Bio Inc.) using specific primers (Table S1) and analyzed with QuantStudio 6 Flex real-time PCR systems (Applied Biosystems, Carlsbad, CA, USA). 

### 2.12. Western Blot Analysis

Whole-cell lysates from iEPCs were prepared using radioimmunoprecipitation assay (RIPA) buffer (150 mM sodium chloride, 1% Triton X-100, 0.5% sodium deoxycholate, 0.1% SDS, and 50 mM Tris, pH 8) containing Halt Protease Inhibitor Cocktail (Thermo Scientific, Rockford, IL, USA) and phenylmethanesulfonyl fluoride (PMSF) (Sigma Aldrich, St. Louis, MO, USA). The lysates were quantitated using the Bradford Reagent (Biorad Inc., Richmond, CA, USA). A total of 30 μg of the lysate was loaded on a 7% sodium dodecyl sulfate-polyacrylamide gel electrophoresis (SDS-PAGE) gel and analyzed by western blot using primary antibodies, anti-*BCL11A* (1:1000 dilution) (Cell Signaling Technologies, Danvers, MA, USA), and anti-actin (1:5000 dilution) (BD Pharmingen, San Jose, CA, USA) and secondary antibodies, anti-mouse IgG HRP (Cell Signaling Technologies, Danvers, MA, USA) and anti-rabbit IgG HRP (Invitrogen Corporation, Camarillo, CA, USA). The signal was detected using the Westar Supernova (Cyanagen, Bologna, Italy) and FluorChemE gel documentation system (Protein Simple, San Jose, CA, USA).

## 3. Results

### 3.1. Generation of iEPCs from PBMNCs

**Figure 1:**
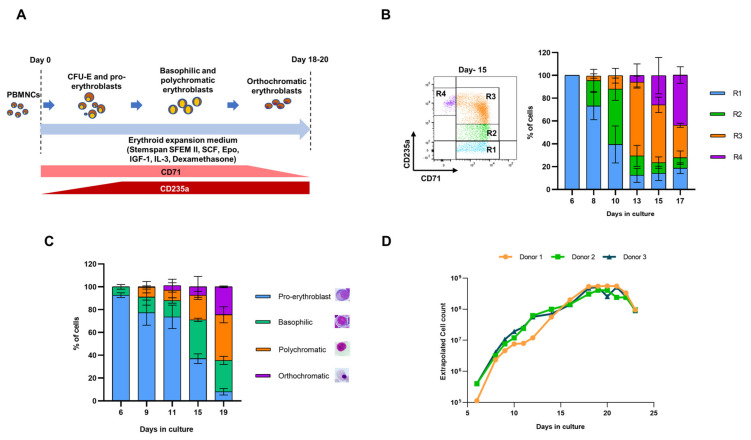
The modified PBEPC culture protocol that yields a high number of early-stage EPCs for a prolonged duration in culture. (**A**) Schematic representation of the erythroid culture protocol of PBMNCs. SCF: stem cell factor, IL-3: interleukin-3, Epo: erythropoietin, IGF-1: insulin growth factor 1. (**B**) (Left) A representative flow cytometry result of CD71 and CD235a expression from day 15 of the culture and designation of R1, R2, R3, and R4 populations. (Right) The percentages of R1, R2, R3, and R4 populations on different days of the culture using the modified erythroid culture protocol. CD71- and CD235a- lymphocytes present until day 9 of the culture are not shown. (**C**) Percentages of the EPCs representing the different stages of erythropoiesis (pro-, basophilic, polychromatic, and orthochromatic erythroblasts) on different days of the erythroid culture. (**D**) The number of erythroid cells generated from 5 × 10^6^ PBMNCs on different days of erythroid cultures. Data from day 6 of the erythroid culture is shown.

In a culture medium containing the supporting cytokines, very early stage EPCs including blast forming unit-erythroid (BFU-E) and colony-forming unit-erythroid (CFU-E) cells present in the PB can be selectively expanded without preselection [36,49,50,51]. During expansion, they also undergo progressive erythropoiesis to form the cells at the later stages of differentiation, pro-, basophilic, polychromatic, and orthochromatic erythroblasts and enucleated reticulocytes, with characteristic changes in cell morphology and expression of surface markers. As HEE immortalizes early-stage proerythroblasts predominantly, a culture protocol favoring slow in vitro differentiation will provide sufficient numbers of proerythroblasts for a prolonged duration in the culture for their transduction with HEE lentiviruses and subsequent immortalization. Therefore, we standardized a modified EPC culture protocol (Figure 1A) using a serum-free medium containing dexamethasone, which has been shown to increase the number of early-stage EPCs in culture by slowing their differentiation to later stages of erythropoiesis [52,53]. We observed CD71^+^CD235a^–^ EPCs, which resemble colony-forming unit erythroid (CFU-E) cells [50,51], emerged on days 6 or 7 of the culture (Figure 1B). In the following two weeks, these cells underwent differentiation to form CD71^high^CD235a^-^ (R1), CD71^high^CD235a^medium^ (R2), CD71^high^CD235a^high^ (R3) and CD71^low^CD235a^high^ (R4) EPC populations (Figure 1B), which represent CFU-E, pro-, basophilic, and polychromatic erythroblasts, respectively [50,51] (Figure 1C). A large number of lymphocytes present in the initial days of culture disappeared due to the selective expansion of EPCs. Starting with 5 × 10^6^ PBMNCs, we could obtain up to 5 × 10^8^ EPCs by day 20 using this protocol (Figure 1D). After day 20, there was a significant reduction in the cell number (Figure 1D) as the medium did not support the proliferation and differentiation of late-stage CD71^low^ CD235a^high^ orthochromatic EPCs. In this protocol, erythroid differentiation was slow, and a significant number of proliferating CFU-E like cells and proerythroblasts were obtained for a long period in the culture (~2 weeks). The CD71^high^ CD235a^−^ (R1) cells ranged from 60–83.6% (73.1 ± 12) on day 8, 15–24% (18.6 ± 4.7) on day-13, and 5.5–9.8% on day 17 (7.6 ± 2.1) (Figure 1B). The CD71^high^ CD235a^low^ (R2) levels ranged from 14–33.1% (22.3 ± 9.7) on day 8, 6.4–22.4% (17 ± 9.2) on day 13, and 2.63–13.8% (9.2 ± 5.8) on day 17 (Figure 1B).

**Figure 2:**
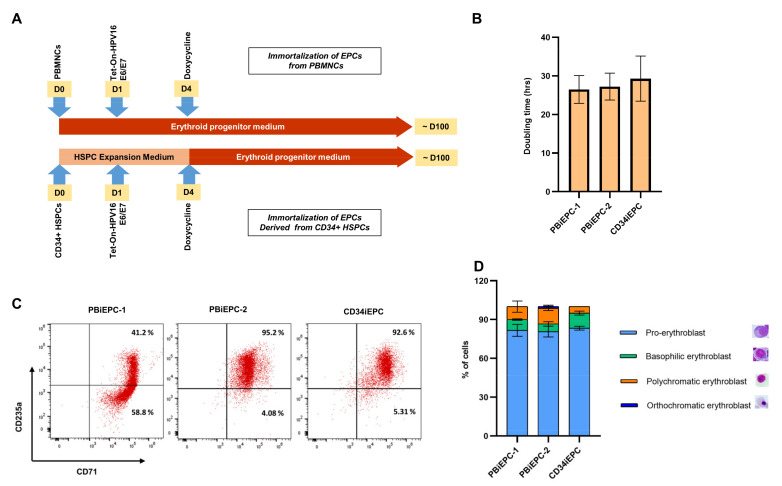
Generation of iEPCs from PBMNCs and CD34^+^ HSPCs. (**A**) Illustration of the steps involved in the generation of iEPCs from PBMNCs (top) and CD34^+^ HSPCs (bottom). (**B**) Graph showing the doubling times of PBiEPC-1 and PBiEPC-2 generated from PBMNCs and CD34iEPC generated from CD34^+^ HSPCs. (**C**) Flow cytometry analysis of the expression of erythroid surface markers, CD71 and CD235a, in the undifferentiated iEPCs. (**D**) Percentages of different types of EPCs present in the undifferentiated iEPCs (data from three different passages are shown).

Since we could obtain a large number of early EPCs (CFU-like cells and proerythroblasts) for two weeks in the culture, and the lentiviral expression of transgenes could be achieved in less than five days after transduction, we decided to test the possibility of immortalizing these cells from the initial days of the culture by HEE lentiviruses. We isolated PBMNCs from two healthy donors, and the cells were transduced with HEE viruses and cultured in EPM for the selective expansion of EPCs (Figure 2A). On day 4, before CD71^+^CD235a^−^ EPCs emerged, the medium was supplemented with dox to induce HEE expression. Flow cytometry analysis of the constitutively expressed hKO1 fluorescent protein from the lentiviral vector showed 30–32% hKO1^+^ cells on day 7 (Figure S1). After 30–35 days, almost all the cells in the culture (>90%) were hKO1^+^CD71^+^CD235a^+^ (Figure S1), which suggested the initiation of immortalization of the transduced cells. After ~100 days, the cells presented the properties of immortalized erythroblasts [19,20,54], a stable doubling time (Figure 2B), and consistent expression of CD71 and CD235a (Figure 2C). We designated these iEPCs derived from peripheral blood as PBiEPC-1 and PBiEPC-2. We observed a difference in the erythroblast stage of these iEPCs; PBiEPC-1 consisted of 79–87% (81.6 ± 4.6) proerythroblasts, 8–9% (8.3 ± 0.5) of basophilic erythroblasts, and 5–13% (10 ± 4.3) of polychromatic erythroblasts, while PBiEPC-2 consisted of 76–84% (80.6 ± 4.1) proerythroblasts, 5–8% (6 ± 1.7) of basophilic erythroblasts, 11–14% (12 ± 1.7) of polychromatic erythroblasts, and 0–2% (1.33 ± 1.15) orthochromatic erythroblasts (Figure 2D). Altogether, these results showed that prolonged maintenance of early-stage EPCs in culture helps generate iEPCs from PB successfully, without using CD34^+^ HSPCs from the donors.

### 3.2. Variegation in HEE Expression Causes Lag in Immortalization of EPCs

Our protocol for the generation of PBiEPCs is suitable for generating cell lines from patients with red cell diseases. Immortalization with stable cell proliferation could be achieved only after ~15–18 weeks (>100 days), although >90% of the cells on day 30 of the culture were hKO1^+^. This significant delay to establish immortalization in EPCs has been previously reported [55]. Analysis of the expression of HEE genes and CD71 and CD235a markers in the cells in the pre-immortalization stage (from the day of transduction until immortalization) could help understand the kinetics of erythroid cell immortalization and in developing efficient and faster protocols for establishing immortalized EPCs.

In a dox-inducible vector, the expression of constitutively expressed genes correlates with the expression of the inducible genes [56]. Therefore, we measured the expression of hKO1 to monitor the relative expression of HEE in the cells undergoing immortalization. On day 35 and day 80 of the pre-immortalized stage, the cells had heterogeneous expression levels of hKO1 (Figure S1A) and CD71 and CD235a (Figure S1B) compared to those from the immortalized stage (Figure 2C). We flow-sorted hKO1^bright^, hKO1^medium^, and hKO1^low^ populations from day 50 of the culture and performed flow cytometry analysis of the percentages of R1, R2, R3, and R4 populations. We found that the hKO1^bright^ population consisted of early-stage EPCs, whereas hKO1^medium^ and hKO1^low^ populations consisted of the late-stage EPCs (Figure S2). The hKO1^bright^ cells were cultured in EPM to understand whether they would achieve stable cell proliferation and immortalization faster due to the high expression of HEE in these cells. However, after two weeks, a large number of CD71^high^ CD235a^high^ and CD71^low^ CD235a^high^ late-stage EPCs were formed in the culture, resulting in a significant reduction in the total cell number. After ~100 days, stable cell proliferation (Figure 2B) and CD71 and CD235a expression (Figure 2C) were attained when the cells with homogenous hKO1 expression (Figure S1) without further transgene silencing and differentiation persisted in culture. 

We concluded from these results that the lag in achieving the immortalization with stable cell proliferation is due to the variegated expression of the lentivirally transduced HEE genes and transgene-silencing in a large number of EPCs during immortalization. The cells with low expression of HEE genes differentiated to form late-stage EPCs, resulting in a reduced cell number and delay in achieving a population of cells with stable proliferation. Immortalization is finally achieved when the cells with high hKO1 expression alone persist in the culture. Although monitoring the transduced cells for high and uniform expression of hKO1 enables identifying cells that are successfully immortalized, we found that the immortalization of EPCs is a slow process requiring a long period of continuous culture of cells.

### 3.3. Generation of an iEPC Line from CD34^+^ HSPCs 

We wanted to compare the kinetics of the immortalization process and the properties of the iEPCs obtained from PB with those from CD34^+^ HSPCs. CD34^+^ cells from a normal donor were transduced with HEE lentiviruses and cultured for five days in the HSPC expansion medium. Subsequently, the cells were cultured in EPM containing dox to induce erythroid differentiation and expression of HEE genes (Figure 2A). CD71^+^CD235a^−^ cells emerged in the culture after 5–6 days, and they differentiated to CD71^+^CD235^low^ and CD71^high^CD235^high^ EPCs gradually (Figure S1A). We observed a similar pattern of immortalization kinetics as observed during the generation of PBiEPCs, a long pre-immortalization stage for ~100 days with a heterogeneous expression of hKO1 and a significant reduction in cell number due to the formation of late-stage EPCs with low HEE expression. The immortalized cells with stable cell proliferation and hKO1 expression were observed from day 120, and this CD34 derived iEPCs was designated as CD34iEPC. Flow cytometry analysis of CD71 and CD235a expression showed that CD34iEPC is similar to PBiEPCs and other previously reported iEPCs [57,58] (Figure 2C). Morphology analysis showed that this cell consisted of 82–85% (83.3 ± 1.5) proerythroblasts and 10–13% (11.6 ± 1.5) of basophilic and 5% polychromatic erythroblasts (Figure 2D).

### 3.4. Differentiation of iEPCs

**Figure 3:**
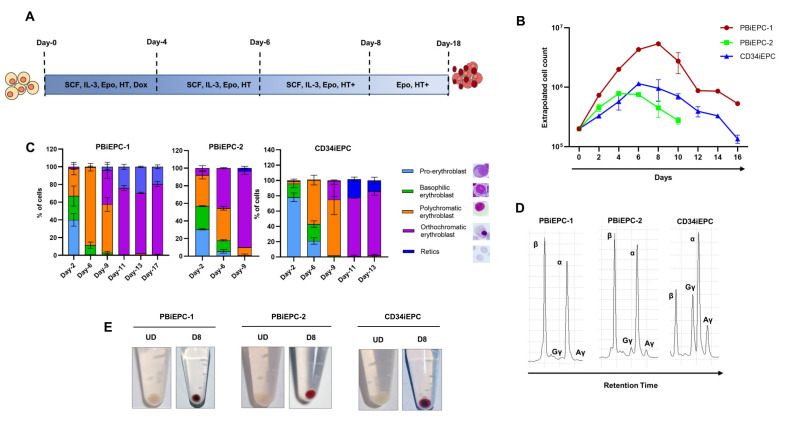
Differentiation of iEPCs. (**A**) Schematic representation of the differentiation protocol for iEPCs. SCF: stem cell factor, IL-3: interleukin-3, Epo: erythropoietin, HT: holotransferrin and dox: doxycycline. HT+ indicates increased holotransferrin concentration in the medium. (**B**) Extrapolated cell count of iEPCs during the different days of erythroid differentiation. (**C**) Morphology of the iEPCs during erythroid differentiation as analyzed using Wright Giemsa staining. (**D**) Representative reverse-phase HPLC analysis of iEPCs showing the contribution of the individual globin chains in the differentiated iEPCs. (**E**) Cell pellet colors of undifferentiated iEPCs (UD) and differentiated iEPCs from day 8 of differentiation (D8).

The iEPCs generated by HEE expression significantly differed in their terminal differentiation potential to form enucleated reticulocytes [55,57,58]. We differentiated PBiEPC-1, PBiEPC-2, and CD34iEPC using the protocol described by Hawksworth et al. [33] (Figure 3A). The differentiation was terminated when a significant reduction in cell numbers and cell death was observed due to the apoptosis of orthochromatic EPCs that failed to enucleate. Although all three cell lines proliferated extensively from day 2 to day 8 of the differentiation, they had a significant difference in their proliferation rate and kinetics of differentiation (Figure 3B,C), which correlated with the stages these iEPCs were immortalized. PBiEPC-1, which is immortalized at an earlier stage of erythropoiesis, had the highest proliferation rate (30-fold on day 8 of differentiation, which gradually decreased to 2-fold on day 16), and PBiEPC-2, which is immortalized at a later stage, had the lowest cell proliferation (Figure 3B). PBiEPC-1 exhibited the slowest differentiation with a significant number of cells in culture for up to 18 days and highest enucleation rate 18–21% (mean = 19.5 ± 2.1) (Figure 3C). Differentiation of CD34iPEC lasted for 13 days, yielding 11–17% (mean = 14 ± 4.2) reticulocytes. PBiEPC-2 survived only for nine days in the differentiation medium, and it had only 2–5% (mean = 3.5 ± 2.1) enucleated cells. The addition of mifepristone, an antagonist of glucocorticoid that induces enucleation in erythroid differentiation [59], did not show any significant change in the enucleation potential of the three iEPCs.

Centrifuged iEPCs produced white pellets before differentiation and red pellets after differentiation, indicating efficient hemoglobin synthesis in these cells upon differentiation (Figure 3E). We measured the expression of genes that were upregulated during erythropoiesis in the iEPCs undergoing differentiation. Hemoglobin genes, *HBB* and *HBA*, were highly upregulated in the cells from day 6 of differentiation compared to the undifferentiated cells (Figure S3). Erythroid transcription factors, *BCL11A*, *GATA1*, and *KLF1*, were also increased significantly after differentiation (Figure S3). HPLC analysis of globin chains showed adult α- and β-globin chains predominantly in PBiEPC-1 and 2. (Figure 3D). However, CD34iEPC had a very high-level expression of Aγ and Gγ globins (Figure 3D). Our data clearly showed a significant difference in the differentiation kinetics and enucleation potential of iEPCs, and they also exhibited a difference in the expression of erythroid proteins such as hemoglobins.

### 3.5. Karyotype Analysis of iEPCs

Similar to the findings in HUDEP2 and BEL-A [54,58], all three iEPCs that we generated had numerical and structural anomalies, which included whole or partial trisomies, chromosome anomalies, translocations, and partial or whole chromosome losses (Table 1) (Figure S4). PBiEPC-1 has a modal chromosome number of 45 (range: 44–45), which was less than 51 (range 49–53) in HUDEP-2 [54] and 48 (range 44–48) in BEL-A [58]. PBiEPC-2 had two predominant clones with chromosome numbers, 48 and 49. CD34iEPC had very complex karyotypes with a modal chromosome number 84 (range: 79–92). We did not observe any significant similarities among the numerical and structural abnormalities among the different iEPCs (Table 1).

### 3.6. Gene Manipulation of PBiEPC-1

Among the three lines we generated, PBiEPC-1 was immortalized at the earliest stage of erythropoiesis with a large number of R1 population cells (Figure 2C), which represent CFU-E cells [51]. It exhibited slow progressive erythroid differentiation, which lasted up to 18 days with all the stages of erythroid differentiation, generating a large number of cells during differentiation. It had fewer chromosomal abnormalities than HUDEP-2 and BEL-A. Therefore, this cell line is the most suitable iEPC line for studying the sequential molecular changes in erythropoiesis after gene manipulation. We performed experiments to illustrate the utility of this cell line for in vitro gene manipulation. We performed knockdown and knockout experiments to downregulate the expression of BCL11A, a protein that represses the expression of γ-globin gene in adult erythroid cells and is considered a therapeutic target for β-hemoglobinopathies [60].

**Figure 4:**
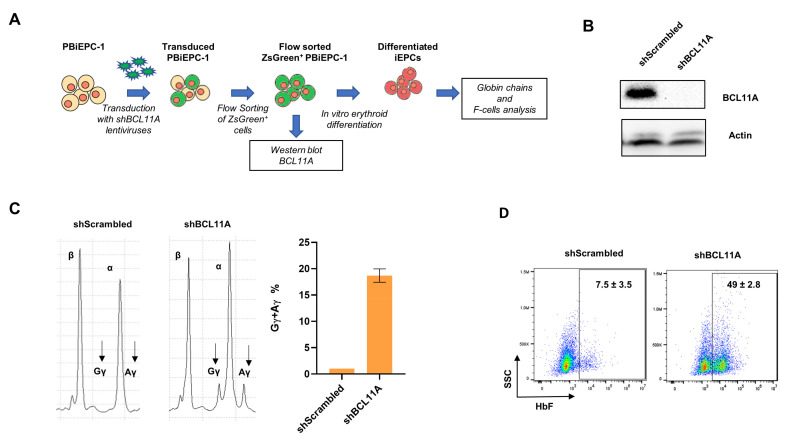
Knockdown of *BCL11A* in PBiEPC-1. (**A**) Schematic representation of the experimental setup for *BCL11A* knockdown in PBiEPC-1. (**B**) Western blot analysis of *BCL11A* knockdown in PBiEPC-1. Data were compared with the cells transduced with the scrambled shRNA. (**C**) (Left) An HPLC chromatogram showing β, α, Gγ, and Aγ globins in the cells transduced with shBCL11A and shScrambled lentiviruses. (Right) Gγ + Aγ% in the differentiated erythroid cells transduced with shBCL11A and shScrambled lentiviruses. (**D**) Flow cytometry analysis of F-cells after knockdown of *BCL11A*. Numbers indicate mean ± standard deviation (SD) from two independent experiments.

**Figure 5:**
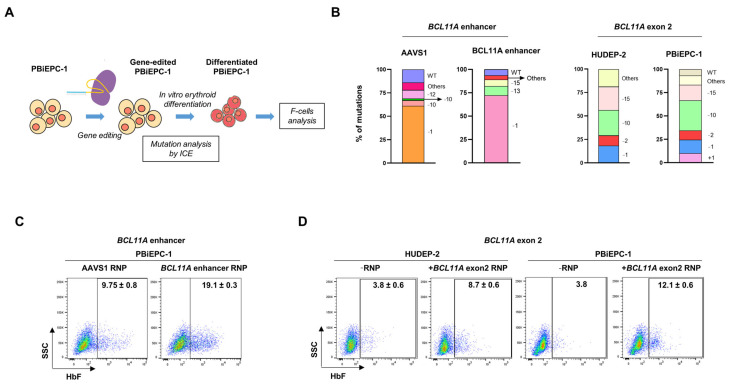
CRISPR-Cas9 mediated gene editing of *BCL11A* enhancer and exon 2 in PBiEPC-1. (**A**) Schematic representation of the CRISPR/Cas9 experiment in PBiEPC-1. (**B**) Inference of CRISPR Edits (ICE) analysis results of gene editing at the *BCL11A* enhancer and exon 2. The percentages of different types of mutations are shown. “+” indicates insertions, and “–“ indicates deletions of nucleotides. “WT” indicates wild type sequence, and “others” indicates rare insertions and deletions. (**C**) Flow cytometry analysis of F-cells in the cells edited at *BCL11A* enhancer and AAVS1 (control genomic region). Numbers indicate mean ± standard deviation (SD) from two independent experiments. (**D**) Flow cytometry analysis of F-cells after editing at *BCL11A* exon 2 in PBiEPC-1 and HUDEP-2. Numbers indicate mean ± standard deviation (SD) from two independent experiments. RNP: ribonucleoprotein complex containing Cas9 protein and gRNA.

For the RNAi experiment, PBiEPC-1 was transduced with pZIP-MND-ZsGreen-Puro-shBCL11A, which expresses an shRNA to downregulate the expression of *BCL11A* (Figure 4A). In the flow-sorted ZsGreen^+^ undifferentiated PBiEPC-1 cells, > 90% reduction in *BCL11A* protein expression was observed (Figure 4B). Globin chain analysis in the differentiated edited cells showed upregulation of γ-globin levels by 17.8–19.6% (18.7 ± 1.2) (Figure 4C). The PBiEPC-1 cells transduced with *BCL11A* shRNA showed ~41% more F-cells than those transduced with scrambled shRNA (49 ± 2.8% vs. 7.5 ± 3.5%) (Figure 4D). For gene editing, we performed CRISPR-Cas9 based approach with the guide RNAs (gRNAs) that target the transcription enhancer and exon 2 of *BCL11A*. PBiEPC-1 was nucleofected with ribonucleoprotein (RNP) complexes containing Cas9 protein and gRNAs, the editing efficiencies at the target regions were determined by ICE analysis [48], and the percentage of F-cells was analyzed in the edited cells after differentiation (Figure 5A). High-efficiency gene-editing of the target genomic regions was observed in PBiEPC-1 at the levels comparable to that obtained with HUDEP-2 cells and at the AAVS1 control genomic site (Figure 5B). The *BCL11A* enhancer edited PBiEPC-1 cells showed a ~10% increase in the F-cells compared to the AAVS1 site edited cells (19.1 ± 0.3% vs. 9.75 ± 0.3) (Figure 5C). The gene-editing efficiency and F-cells% in the *BCL11A* exon 2 edited PBiEPC-1 were compared with those from HUDEP-2 cells edited at the same locus. Similar efficiencies of gene editing at the *BCL11A* exon 2 were observed in the two cell lines (>95%) (Figure 5B). In the *BCL11A* exon-2 edited PBiEPC-1 cells, the F-cells increased by ~8% compared to the unedited PBiEPC-1 cells (12.1 ± 0.6% vs. 3.8 ± 0) while in the edited HUDEP-2 cells, there was ~5% increase in the F-cells (8.7 ± 0.6% vs. 3.8 ± 0.6%) (Figure 5D). These results indicate that the PBiEPCs are amenable for gene manipulation, and the generation of such cell lines from patients with erythroid diseases is a valuable tool for functional studies of the genes of interest.

## 4. Discussion

There is a huge demand for models to study normal and disease erythropoiesis. Although erythroid cells can be generated by ex vivo differentiation of HSPCs, this approach has a limitation of restricted expansion of the cultured erythroid cells resulting in repeat collections of HSPCs for obtaining the substantial number of cells for performing experiments. Differentiation of iPSCs provides a sustainable source of erythroid cells, but they have poor yield, incomplete differentiation, and predominant fetal/embryonic protein expression [13,14,15,16,17]. Recent advances in the immortalization methods have generated iEPCs, which can mimic human erythropoiesis efficiently. HUDEP-2 [19] and BEL-A [20] generated by lentiviral transduction of HEE genes in cord blood and adult CD34^+^ cells have been extensively characterized for their erythroid progenitor properties, including erythroid differential potential. The method used for the generation of these iEPCs is suitable for creating an iEPC cell bank from patients with erythroid diseases for disease modeling and potential drug screening [34]. However, the major challenge for creating an iEPC bank using this method using HEE lentiviral vectors for immortalization is the requirement of bone marrow aspirates from the patients or treatment with drugs such as granulocyte-macrophage-colony-stimulating factor (GM-CSF) to obtain a sufficient number of CD34^+^ HSPCs. Although cord blood HSPCs are easier to obtain, it is uncertain whether the newborns have any red cell diseases. Other limitations of the erythroid cells derived from cord blood CD34^+^ cells have a fetal rather than adult phenotype, and they have low terminal differentiation potential [61,62].

Using a protocol that facilitated the expansion and maintenance of early-stage EPCs and lentiviral transduction of HEE genes, we could generate iEPCs from PB. The PBiEPCs were similar to the previously reported iEPCs, HUDEP-2 and BEL-A, generated by the expression of HEE genes, with respect to their morphology, expression of erythroid-specific surface markers, stable cell proliferation, and the potential to differentiate to late-stage progenitors and enucleated reticulocytes [58]. The enucleation rates of PBiEPCs were ~10% more than HUDEP-2 [19], the most extensively used iEPC line in the literature, and comparable to the recently reported BEL-A cell line [20].

The fact that we could generate PBiEPCs from the few very early stage PB-EPCs suggests that our protocol is highly suitable for generating erythroid cell lines from patients with red cell disorders, without the necessity of HSPCs from the patients. Diseases that cause defective erythropoiesis like CDA and those that cause hemolytic anemias by mutations that affect the red cell membranes could be effectively studied using PBiEPCs derived from the patients with these diseases. As the PBiEPCs express adult hemoglobin, a bank of these cell lines can be generated from a large number of patients with β-thalassemia and sickle cell disease, which will enable studies to analyze the molecular basis of the phenotypic heterogeneity in these diseases. The PBiEPCs generated from patients can also be used for drug screening and identification of new therapeutic targets using high throughput analysis involving RNAi and CRISPR libraries.

As iEPCs can be maintained in culture as early erythroid progenitors and can be induced to differentiate to the cells of late stages of erythropoiesis, they have also been extensively used for studying the mechanisms of human erythropoiesis [63,64] and for identifying genetic factors involved in globin gene switching [26,28,65,66,67] by genetic manipulations of these cells by RNAi [20,67,68] and gene editing [23,26,28,69,70]. Genetic mutations were recently introduced in the genes associated with erythroid diseases to generate disease models [71,72]. Our PBiEPCs could also be efficiently transduced with lentiviral vectors for gene knockdown and gene-edited using CRISPR/Cas9 for studying the functions of the target genes.

It is important to note that the iEPCs generated by HEE expression have a difference in the erythroid stage at which they are immortalized and in their terminal differentiation potential. It has been reported earlier that even iEPCs generated in replicates from the same donor can exhibit differences in their properties [55]. The previously described iEPCs, HUDEP and BEL-A, significantly differed in the immortalization stage and differentiation potential. The PBiEPC-1 line, which was immortalized at a very early stage of erythropoiesis, yielded a large number of terminally differentiated cells with enucleation similar to BEL-A. However, the other two cell lines had poor cell survival and enucleation potential. This technical challenge is an important factor to consider when a bank of iEPCs is generated for disease modeling. Immortalization of erythroid progenitors is a very long process, and we found that this was primarily because of the expression variegation and transgene-silencing of the lentivirally expressed HEE genes. A lentiviral vector containing robust promoters, which do not exhibit significant expression variegation, for the constitutive and inducible expression of reverse tetracycline transactivator (rtTA) and HEE, respectively, may facilitate faster immortalization. A major drawback of the immortalization of erythroid cells is that they have chromosome abnormalities. However, they do not seem to affect the differentiation potential of iEPCs. It is not clear whether these chromosome abnormalities also contribute to the immortalization of erythroid cells.

In conclusion, our results showed that iEPCs could be generated successfully from PB-EPCs without the use of CD34^+^ HSPCs. PBiEPC-1 is superior to the HUDEP-2 line, which is currently being extensively used by researchers. This cell line can be easily gene manipulated for the evaluation of genes involved in erythropoiesis. Generation of such lines from patients with red cell diseases will help develop a cell bank of iEPCs for disease modeling and drug screening.

## Figures and Tables

**Table 1:** Karyotypes of the iEPCs on different days of culture.

Cell Line	Days	Karyotype
PBiEPC-1	162	45,XY,-10,der(10),add(21)(q22)[20]
	169	45,XY,-10,der(10),add(21)(q22)[18]
PBiEPC-2	174	48,XY,+8,-18,+19,+21[10]/49,XY,+8,del(18)(p11.2),+19,+21[7]
	181	44~48,XY,-Y,+8,-18,+19,+21[cp6]
CD34iEPC	143	80~87<4n>,XXX,der(X),-3,add(4)(p13),del(4)(q21),der(4;11)(p13;q23),der(5;11)(q35;q23),-7, add(7)(p14), der(8;15)(p23;p10),-9, add(9)(q34),der(10),-12, der(13;15)(p10;p10),-14,-17, i(17)(q10), add(21)(p10), add(21)(q21), +mar[cp20]
	157	79~92<4n>,XXX,der(X),-3,add(6)(p23),-7,t(7;10)(q32;q24),add(8)(p27), der(8),-9, -10,-11,-11,-12, der(13), -14,-15,der(15),-16,der(17),i(17)(q10),der(21),+mar,+mar[cp20]

## Data Availability

The data presented in this study are available in the article and Supplementary Materials.

## Supplementary Materials

The following are available online at https://www.mdpi.com/2073-4409/10/3/523/s1, Figure S1: Expression of hKO1 fluorescence protein at different stages of immortalization, Figure S2: Percentages of R1, R2, R3, and R4 EPC populations in the flow-sorted hKO1bright, hKO1medium, and hKO1low cells from the pre-immortalized stage, Figure S3: Gene expression analysis of erythroid genes in the iEPCs by real-time PCR, Figure S4: Representative karyotypes of iEPCs on different days after immortalization, Table S1: Details of the oligos used in the study.
