# Supplementary material for: Direct Generation of Immortalized Erythroid Progenitor Cell Lines from Peripheral Blood Mononuclear Cells
Source: Cells. 2021 Mar 1;10(3):523. doi: 10.3390/cells10030523 (PMC7999632; doi:10.3390/cells10030523)
Supplement: Supplementary file 1 [file cells-10-00523-s001.pdf]

Supplementary Figures and Tables.

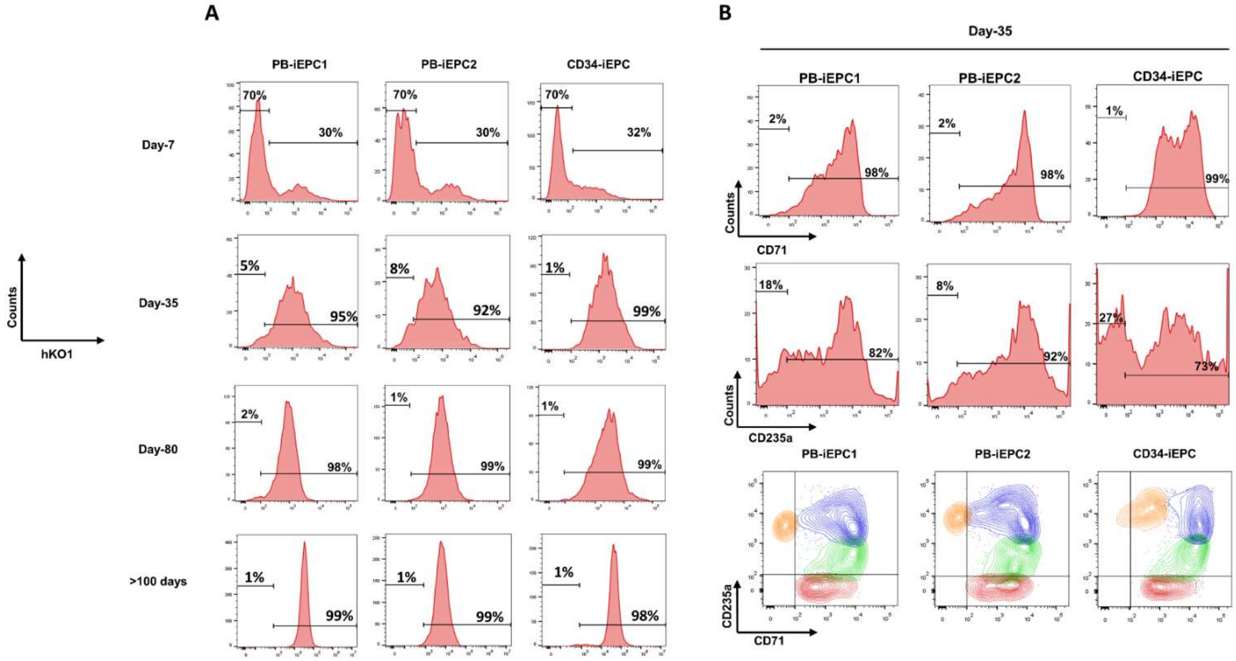

Figure S1. Expression of hKO1 fluorescence protein at different stages of immortalization. (**A**) hKO1 expression after 7 days of transduction, ~35 days in EPM, 80 days in EPM and after completion of immortalization (> 100 days), (**B**) Flow cytometry analysis of CD71 and CD235a expression 35 days after transduction of HEE lentiviruses.

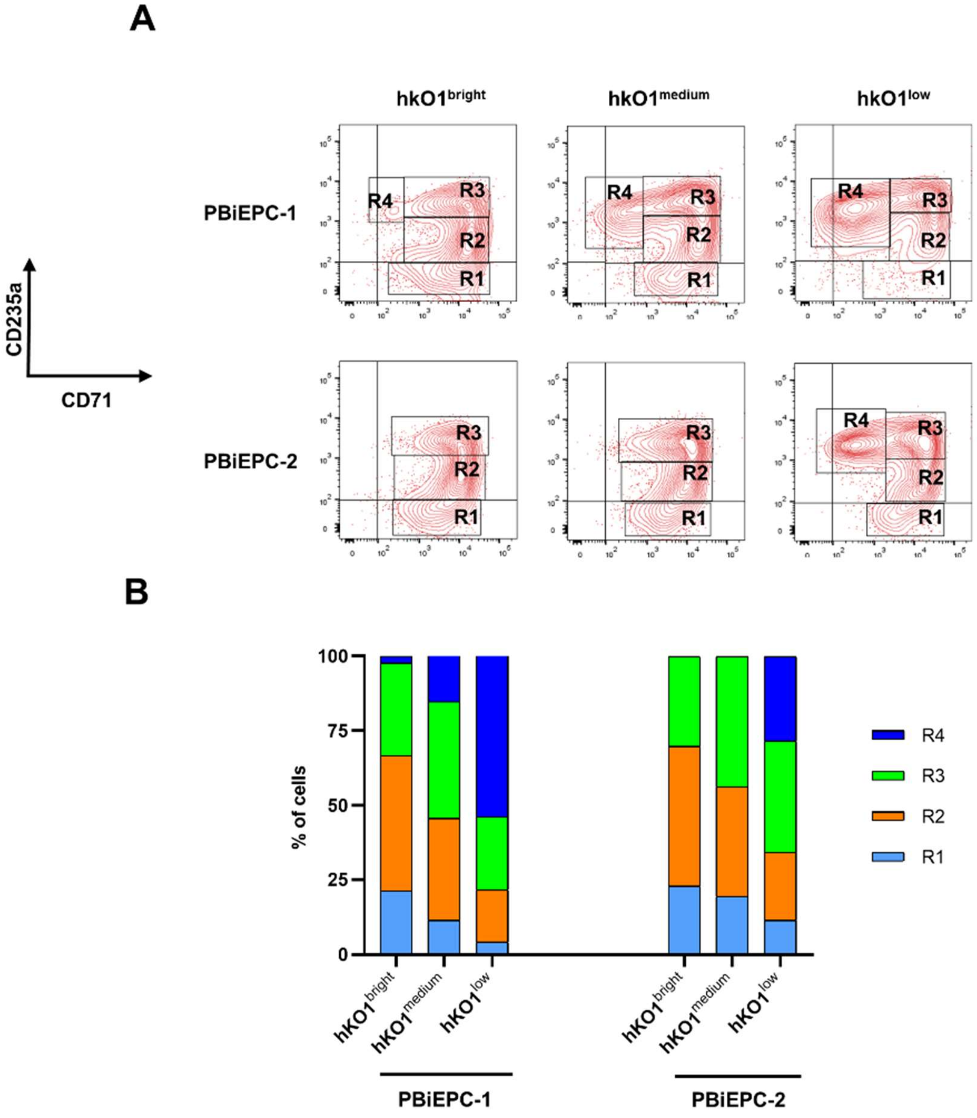

Figure S2. Percentages of R1, R2, R3 and R4 EPC populations in the flow-sorted hKO1^{bright}, hKO1^{medium} and hKO1^{low} cells from the pre-immortalized stage. (**A**) Distribution of R1, R2, R3 and R4 populations identified based on CD71 and CD235a expression in the hKO1^{bright}, hKO1^{medium} and hKO1^{low} cells from the pre-immortalized stage. (**B**) Graphical representation of the percentages of R1, R2, R3 and R4 populations in the flow-sorted hKO1^{bright}, hKO1^{medium} and hKO1^{low} iEPCs.

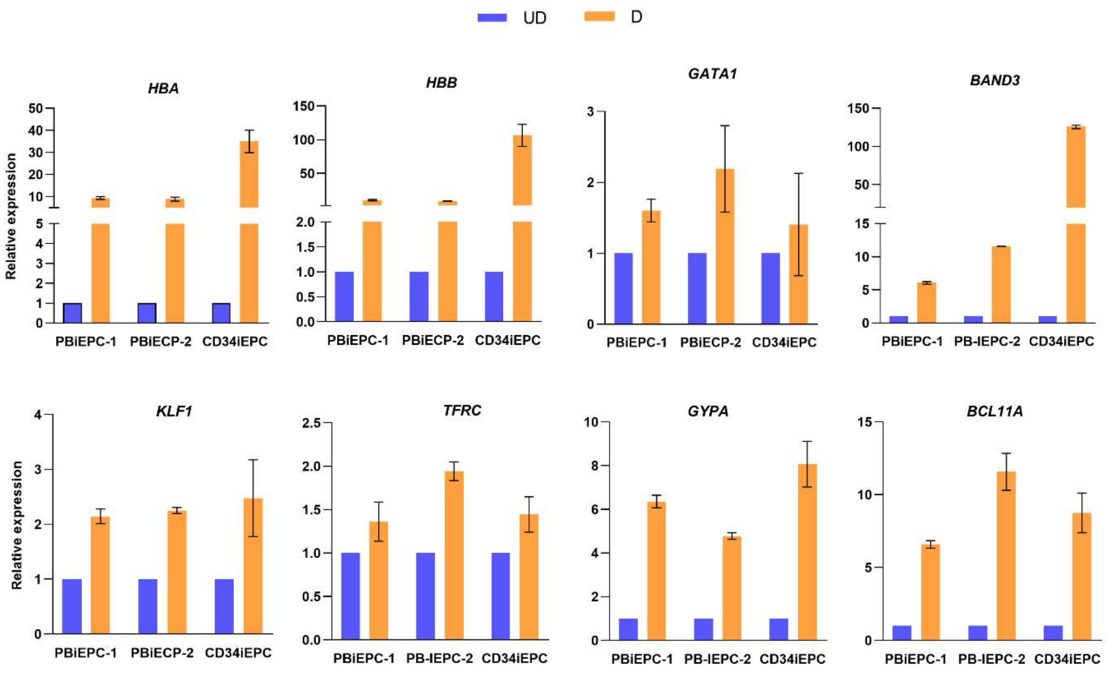

Figure S3. Gene expression analysis of erythroid genes in the iEPCs by real-time PCR. UD and D indicate the results from the undifferentiated and the differentiated (day-6) iEPCs, respectively. Fold change in expression in the differentiated cells was calculated by normalizing with the values from undifferentiated cells.

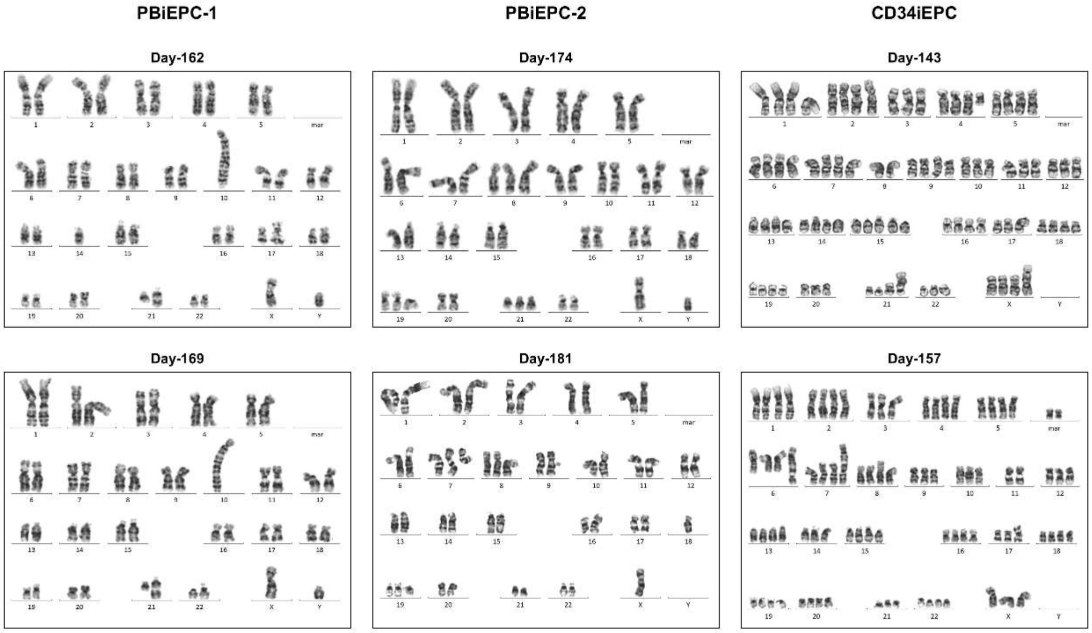

Figure S4. Representative karyotypes of iEPCs on different days after immortalization.

Table S1. Details of the oligos used in the study.

Target	Oligo Name	Sequence (5'—3')
	**PCR Primers**	
HBA	HBA -*Forward*	TCAAGCTCCTAAGCCACTGC
	HBA -*Reverse*	AGAAGCCAGGAACTTGTCCA
HBB	HBB -*Forward*	CAGGCTGCTGGTGGTCTAC
	HBB -*Reverse*	GCCATGAGCCTTCACCTTAG
BCL11A	BCL11A -*Forward*	ATGCGAGCTGTGCAACTATG
	BCL11A -*Reverse*	GTAAACGTCCTTCCCCACCT
GATA1	GATA1 -*Forward*	CACTGAGCTTGCCACATCC
	GATA1 -*Reverse*	ATGGAGCCTCTGGGGATTA
KLF1	KLF1 -*Forward*	ACACCAAGAGCTCCCACCT
	KLF1 -*Reverse*	GTAGTGGCGGGTCAGCTC
TFRC	TFRC -*Forward*	TCAGAGCGCGGGATATCG
	TFRC -*Reverse*	TGAACTGCCACACAGAAGAACA
GYPA	GYPA -*Forward*	CGGTATTCGCCGACTGATAAA
	GYPA -*Reverse*	AAAGGCAGTCTGTGTGAGGT
BAND3	BAND3 -*Forward*	TCTTCAGGAACGTGGAGCTT
	BAND3 -*Reverse*	CCTCATCAAAGGTTGCCTTG
BCL11A (enhancer)	*BCL11A enh -Forward*	TCAAACCACAGGGATCACAA
	BCL11A enh -Reverse	AGAGAGCCTTCCGAAAGAGG
BCL11A (exon- 2)	*BCL11A exon- 2 -Forward*	GAAGGGGAAGGTGGCTTATC
	BCL11A exon- 2 -Reverse	ATTTGTTGCTGTGGGTGTGT
AAVS1	*AAVS1 -Forward*	GAGATGGCTCCAGGAAATGG
	AAVS1 -Reverse	ACCTCTCACTCCTTTCATTTGG
	gRNA sequences	
BCL11A exon 2	*BCL11A exon 2-sgRNA*	AAGAAUGGCUUCAAGAGGCU
BCL11A enhancer	*BCL11A enhancer-sgRNA*	CUAACAGUUGCUUUUAUCAC
